# Ameliorative Effects of Antibiotic-, Probiotic- and Phytobiotic-Supplemented Diets on the Performance, Intestinal Health, Carcass Traits, and Meat Quality of *Clostridium perfringens*-Infected Broilers

**DOI:** 10.3390/ani10040669

**Published:** 2020-04-12

**Authors:** Elsayed O.S. Hussein, Shamseldein H. Ahmed, Alaeldein M. Abudabos, Gamaleldin M. Suliman, Mohamed E. Abd El-Hack, Ayman A. Swelum, Abdullah N. Alowaimer

**Affiliations:** 1 Department of Animal Production, College of Food and Agriculture Sciences, King Saud University, P.O. Box 2460, Riyadh 11451, Saudi Arabia; aabudabos@ksu.edu.sa (A.M.A.); gsuliman@ksu.edu.sa (G.M.S.); aswelum@ksu.edu.sa (A.A.S.); aowaimer@ksu.edu.sa (A.N.A.); 2 Department of Basic Sciences, College of Veterinary Medicine, Sudan University of Science and Technology, Khartoum 12942, Sudan; shamshahmed@yahoo.com; 3 Poultry Department, Faculty of Agriculture, Zagazig University, Zagazig 44511, Egypt; dr.mohamed.e.abdalhaq@gmail.com; 4 Department of Theriogenology, Faculty of Veterinary Medicine, Zagazig University, Zagazig 44511, Egypt

**Keywords:** probiotic, *Clostridium perfringens*, phytobiotic, broiler

## Abstract

**Simple Summary:**

Necrotic enteritis is considered the most important economic problem for the poultry industry due to the sudden death rates of up to 50%. However, there is limited information concerning the ameliorative role of probiotic and/or phytobiotic compounds in the prevention of *Clostridium perfringens* infections in broilers. Hence, this trial is conducted to evaluate the influence of some antibiotic, probiotic and phytobiotic compounds (Maxus, CloStat, Sangrovit Extra, CloStat + Sangrovit Extra, and Gallipro Tect) on the growth performance, carcass traits, intestinal health, and meat quality of broiler chicks. The obtained in vivo results highlight that a probiotic- and/or phytobiotic-supplemented diet has many positive effects on the performance, organ weight, and meat quality of broilers. Besides, a notable reduction in the lesion score is observed with a combined probiotic and phytobiotic diet.

**Abstract:**

The poultry industry needs efficient antibiotic alternatives to prevent necrotic enteritis (NE) infections. Here, we evaluate the effects of probiotic and/or prebiotic dietary supplementation on performance, meat quality and carcass traits, using only an NE coinfection model, in broiler chickens. Three hundred and twenty-four healthy Ross 308 broiler chicks are allocated into six groups. Taking a 35 d feeding trial, the chicks are fed a basal diet with 0.0, 0.1, 0.5, 0.12, 0.5 + 0.12, and 0.2 g Kg^−1^ for the control (T_1_), Avilamycin (Maxus; T_2_), live probiotic (CloStat (*Bacillus subtilis*);T_3_), natural phytobiotic compounds (Sangrovit Extra (sanguinarine and protopine); T_4_), CloStat + Sangrovit Extra (T_5_), and spore probiotic strain (Gallipro Tect (*Bacillus subtilis* spores); T_6_) treatments, respectively. Occurring at 15 days-old, chicks are inoculated with *Clostridium perfringens.* The obtained results reveal that all feed additives improve the performance, feed efficiency, and survival rate, and reduces the intestinal lesions score compared with the control group. The T_6_ followed by T_3_ groups show a significant (*p* < 0.05) increase in some carcass traits, such as dressing, spleen, and thymus percentages compared with other treatments. Also, T_5_ and T_6_ have significantly recorded the lowest temperature and pHu values and the highest hardness and chewiness texture values compared to the other treated groups. To conclude, probiotics combined with prebiotic supplementation improves the growth, meat quality, carcass characterization and survival rate of NE-infected broiler chickens by modulating gut health conditions and decreasing lesion scores. Moreover, it could be useful as an ameliorated NE disease alternative to antibiotics in *C. perfringens* coinfected poultry.

## 1. Introduction

*Clostridium perfringens* is a Gram-positive bacterium which is common within ecosystems and healthy intestinal microflora [1]. *C. perfringens* is responsible for several diseases in humans, wildlife, and farm animals [2], and is the leading cause of necrotic enteric (NE) disease in farm animals, especially in poultry [3]. Regarding birds, NE disease is caused by specific strains of *C. perfringens*. It costs the global poultry industry over two billion dollars annually, mainly due to the high costs of antibiotics and inactive feed conversion [4,5]. Several studies have reported that *C. perfringens* bacteria could produce more than 15 different toxins [6,7]. Although all *C. perfringens* types can induce α-toxin [3], this kind of toxin causes serious enteric and intestinal diseases in animals and humans [8]. Infected birds show severe lesions of the jejunum and ileum, with the small intestine presenting a degenerated mucosa and is distended by gases produced by *C. perfringens* [9]. Signs of infection include depression, reduced mobility, and diarrhoea, which is the most visibly obvious symptom [10].

Several strategies are commonly used to alleviate the symptoms of enteritis in broilers, including the use of probiotics [11]. It has been recognized that administering antibiotics as feed additives can avoid mortalities induced by NE [12]. Avilamycin is an antibiotic of the oligosaccharides family and is widely active against Gram-positive bacteria [13]. Moreover, Avilamycin has been shown to have potent bactericidal effects on *C. perfringens* in vitro [11]. Similar results were found by Paradis et al. [11] and Mwangi et al. [14], who reported linear relationships between the level of Avilamycin in the feed and a reduction in NE mortalities, NE lesion scores, and intestinal *C. perfringens* count. 

A probiotic is a live microbial feed supplement which beneficially affects the host animal by improving its intestinal microbial balance [15,16,17]. Probiotics can interact with the host to improve immunity and intestinal morphology or stimulate the metabolism, thus reducing the risk of infection by opportunistic pathogens [18]. Probiotic bacteria also have been shown to produce molecules with antimicrobial activities, such as bacteriocins, which target specific pathogens or inhibit the adhesion of pathogens or the production of pathogenic toxins [19,20]. Moreover, beneficial bacteria can act as competition against pathogenic strains within the host [21]. A large number of studies have described the isolation of some strains belonging to the genera *Bacillus* and *Lactobacillus*, which exhibit anti*-C. perfringens* activity in vitro [22,23]. The supplementation of animal feed with *Bacillus* spores (*B. licheniformis*) also was tested and proven to be an efficient alternative to antibiotics when used in larger amounts and for longer periods [24]. When 20 day-old chicks, inoculated with low amounts of *C. perfringens*, were given a single dose of 10^9^ *B. subtilis* spores, the colonization and persistence of *C. perfringens* were abolished. However, the *B. subtilis* strain alone was unable to affect *C. perfringens* in vitro [25]. Also, Sokale et al. [26] reported that using *Bacillus subtilis* as a feed supplement in broiler chicks increased the performance and reduced mortality in the chicks treated with *C. perfringens*. The supplementation of *B. subtilis* not only controlled *C. perfringens*-induced NE, but also improved the intestinal health of the broilers [27].

Several natural products such as herbs, spices and essential oils are categorized under the term botanic, phytobiotic or phytogenic compounds [28,29,30]. Phytobiotics are well identified for their antibacterial and pharmacological effects and, thus, are commonly used in broiler feed as growth promoters and alternative medicines [17]. A huge number of in vitro and in vivo studies have approved a varied range of activities for phytobiotics in poultry nutrition, like stimulation of feed intake, or antimicrobial, coccidiostatic, anthelmintic and immunostimulating actions [31]. Abudabos et al. [32] reported that using Sanguinarine as a feed supplement in broiler chicks (Ross 308) challenged with *Clostridium perfringens* enhanced performance, carcass traits and some blood biochemical parameters. Also, El–Sheikh et al. [33] informed that the prebiotic supplemented (Sanguinarine) diet could be an effective treatment alternative to antibiotics for controlling necrotic enteritis diseases in broilers. Diverse types of additives include phytobiotics (primary or secondary components of plants that contain bioactive compounds that exert a positive effect on the growth and health of animals) which could be a beneficial strategy that regulates the gastro-intestinal microbial community and improve broiler health [34].

Found in the literature, the positive effects of both *B. subtilis* and phytobiotic compounds have been reported. However, their synergistic effects have not been described yet. The present study aims to evaluate the ameliorative effects of probiotic and phytobiotic compounds alone or in a combined form of two different types of *B. subtilis* on the performance, carcass traits, meat measurements, and intestinal health of *C. perfringens*-infected broilers during the starter and finisher phases.

## 2. Materials and Methods 

The experiment was performed in cage pens under similar managerial and hygienic conditions in an environmentally controlled poultry unit at the Animal Production Department, College of Food Science and Agriculture Science, King Saud University. All protocols were chosen according to the experimentation guidelines of the Animal Use Ethics in Research Committee of King Saud University (approval number: SE-19-150).

### 2.1. Experimental Design and Feeding Regime

Altogether, 324 day-old broiler chicks (Ross 308 strain) were randomly distributed into six groups. Each group contained nine replicates, with six birds per replicate, and were used for a 35 d feeding trial period. Each group was assigned to one of the following dietary treatments: T_1_; basal diet (control), T_2_; diet supplemented with 0.1 g kg^−1^ of Maxus (antibiotic), T_3_; diet supplemented with 0.5 g kg^−1^ of Clostat (natural probiotic strain), T_4_; diet supplemented with 0.12 g kg^−1^ of Sangrovit (phytobiotic), T_5_; diet supplemented with 0.5 g kg^−1^ of Clostat combined with 0.12 g kg^−1^ of Sangrovit, and T_6_; diet supplemented with 0.2 g kg^−1^ of Gallipro Tect (spore probiotic). The chicks were fed a starter diet between days 0–14, which was then switched to a grower diet between days 15–35 (Table 1). After 14 d, all treated groups were inoculated with *C. perfringens* bacteria. Birds were fed ad libitum, and water was available at all times during the experimental period.

Maxus was manufactured by BIOFERM CZ, spol. Sro. Banskobystrická 461, 621 00 Brno-Řečkovice a Mokrá Hora, Czechia, as a source of Avilamycin antibiotics (each 1000 g, containing 100 g of Avilamycin) in feed diets, while, CloStat products were manufactured by KERMIN Ind., Inc., 2100 Maury Street Des Moines, IA 50317 USA (each 1 g, containing 2 × 10^7^ CFU/g *Bacillus subtilis*). The Sangrovit Extra used phytobiotic compounds (extracts of Benzophenanthridine alkaloids (sanguinarine) and protopine) produced by Albitalia s.r.L., Co., Milano, Italy. Gallipro Tect were used as a source of a highly-selected strain (DSM17299) of *Bacillus subtilis* (*B. subtilis* 4 × 10^9^ CFU/g DSM 17299), and was provided by Boege Alle Co., Hoersholm, Denmark.

### 2.2. Challenge with Clostridium perfringens Bacteria

The *C. perfringens* challenge model was performed as described by Prescott [35]. All treated groups received a *C. perfringens* challenge at a rate of 4 × 10^8^ CFU g^−1^ via oral gavages on day 14, as recommended by Olkowski et al. [36], using the defined B positive bacteria *C. perfringens* isolated from a local farm. The identified bacterium was previously confirmed to be sensitive to antibiotics (Avilamycin) using the broth dilution method (MIC testing) [37]. An inoculum was equipped to contain nearly 10^8^ cells of *C. perfringens* per ml and was processed at a ratio of 1:1.5 ration-to-broth [11]. The challenge feed was mixed with the treatment diet. After the administration of the challenged material, dead birds were counted as study mortalities. These birds underwent a necropsy evaluation to estimate the cause of death/disease.

### 2.3. Growth Performance Parameters

During the starter (0–14-days-old) and finisher (15–35-days-old) periods, the growth and feed efficiency parameters of the broiler chicks were estimated. The daily feed intake was calculated by subtracting the quantity of feed rejected from the feed offered. Additionally, live body weight was estimated at biweekly intervals, while the final body weight and total feed consumption were determined at the end of each trial period. The body weight gain (BWG) was measured by calculating the difference between the live body weight and final body weight for each trial period. The feed conversion ratio (FCR) was computed for each group, as mentioned by Abudabos et al. [32], using the following formula:

FCR = Feed intake/Weight gain(1)

Meanwhile, the production efficiency factor (PEF) was calculated as suggested by Griffin [38], using the following formula:

PEF = (Livability × Live weight (kg))/(Age in days × FCR) × 100(2)

During the feeding trial, the number of deaths was counted to calculate the survival rate, as mentioned by Hussein et al. [39], using the following formula:

Survival rate (SR)% = (the number of the surviving broilers / the initial number of broilers) × 100(3)

### 2.4. Carcass Measurements and Lesion Scores

Ten broiler chicks were randomly collected from each group to estimate the organ weights (heart, gizzard, liver, bursa, spleen, thymus, small and large intestine, and ceca). Chicks were weighed before sacrifice. All internal organs were weighed immediately after slaughter. The gizzard was weighed after its content was removed. The small intestine was measured by determining the distance between the site of the duodenum emergence from the gizzard and the beginning of the ceca. The organ weight was calculated relative to the live body weight. 

According to the Hofacre et al. [40] procedure, two birds per pen were examined for gross intestinal lesions. The characteristic of necrotic enteritis was defined using the description of the Long et al. [41] study. The lesion scores were outlined as follows: 0 = none, 1 = mild, 2 = moderate and 3 = marked (severe) [42].

### 2.5. Meat Characteristics

At the end of the finisher period (35-days-old chicks), three birds were randomly selected per pen to estimate the meat characteristics followed by the Chen et al. [43] method. After euthanasia, the jugular vein was cut, the feathers, heads, and shanks were removed, and the remaining carcasses were dissected. The left and right breast from each bird were used for the quality measurements. The breast samples were stored at −80 ℃ until further analysis. At the time of the analysis, frozen muscles were thawed overnight in a chiller at 4 ℃. 

The pH of the breast muscle was measured twice (after slaughtering and 24 h postmortem), using a microprocessor pH-Meter (Model PH 211, Hanna Instruments, Padova, Italy). The core temperature in the breast muscle was measured after slaughtering with a portable digital thermocouple (Eco Scan Series, Temp JKT, Eu tech Instruments, 7 Gul Circle, level 2M, Keppel Logistic Building, Singapore 629563). The colour values of the CIELAB Color System (L*(lightness), a* (redness), and b* (yellowness)) were determined for the breast muscles 15 min and 24 h after slaughtering, using a Chroma meter (Konica Minolta, CR-400-Japan) following the method used by Castellini et al. [44]. 

The myofibril fragmentation index (MFI) of the breast muscle was determined by multiplying the absorbance value at 540 nm, as described by Culler et al. [45]. The water-holding capacity (WHC) was determined based on the technique described by Hamm [46] and following the modification performed by Wilhelm et al. [47], using the following equation:

WHC = 100 − [(Wi − Wf/Wi) × 100](4)

where, Wi and Wf are the initial and final sample weights, respectively. 

The drip loss (DL) was determined as a percentage based on the initial sample weight. The cooking loss (CL) was determined as the difference between the initial and final weights. Then, cooked samples were used to evaluate the shear force according to the procedure described by Wheeler et al. [48], under a 200 mm/min crosshead speed. The texture profile analysis (TPA) values were estimated using a Texture Analyzer (TA–HD–Stable MicroSystems, Golborne, Warrington WA3 3GR, England) equipped with a compression-platen attachment. The variables determined included the hardness (maximum force needed to compress the sample), cohesiveness (ratio between the total energy required for the first and second compression), springiness (the ability of a sample to recover to its original form after the removal of the compressing force), and chewiness (a resultant of springiness × hardness × cohesiveness).

### 2.6. Statistical Analysis

The data underwent a one-way ANOVA using a completely randomized design. Before analysis, the data were examined for normality of distributions and homogeneity of variance. Percentage data were subjected to arcsine transformation before analysis. SPSS 22 analysis software was used for all statistical analyses. Data are expressed as the mean ± SEM, with a statistical significance level of *p* ≤0.05. Significant differences between means were determined using Duncan’s Multiple Range [49]. 

## 3. Results

### 3.1. Growth Performance and Intestinal Lesions Score

The effects of some feed additives on the total feed intake (TFI), body weight gain (BWG), feed conversion ratio (FCR), and protein efficiency ratio (PEF) of broilers in the starter and finisher periods are shown in Table 2. During the starter period, our results revealed no significant differences between all treatment and non-treatment groups. T_4_ produced the lowest TFI values, while T_3_ and T_1_ exhibited the highest. Regarding the FCR values, both the T_4_ and T_5_ treatments had lower values than those of the other treatments. The dietary supplementation of probiotic bacteria increased the PEF (except for T_3_) compared with the control group.

During the finisher period, all experimental additives significantly increased (*p* < 0.01) the FBW, BWG, FCR, PEF, survival rate (SR), and decreased the intestinal lesions score compared with those of the control group (Table 2). However, the TFI was not significantly affected by the supplemented diets. The T_3_, T_4_ and T_5_ groups had higher BWG values compared with those of the other groups. Additionally, the T_3_-treated group exhibited the best FCR values, while the different treatment groups produced intermediate FCR values. Regarding the PEF values, compared with the control, all experimental additives were significantly enhanced (*p* < 0.05). The SR (%) and lesions score were enhanced considerably by the dietary treatments at 15–35 days of age (Table 2). 

### 3.2. Carcass Measurements

The effects of supplemented diets on carcass traits after a 35-day feeding trial are presented in Table 3. Regarding the T_6_ treatment group, the dressing percentage showed significant (*p* < 0.05) increases. Also, the T_1_ and T_3_ treatment groups recorded the highest significant thymus values, while the lowest values were recorded for T_4_. Meanwhile, the highest significant (*p* < 0.05) spleen weight percentage value was recorded in the T_5_ group.

### 3.3. Meat Characteristics

The effects of feed additives on broiler meat quality parameters are presented in Table 4. The T_3_ and T_5_ treatments resulted in the lowest pHi values, with the highest values observed in T_2_. Conversely, the pHu was significantly reduced by the treatments, with the lowest values recorded in T_5_ and T_6_. Furthermore, the Temperature was significantly reduced in T_6_ followed by T_5_-treated groups compared with other treated groups. However, no significant differences were demonstrated among the other treatment groups concerning the colour component after slaughter (L15 and b15) and 24 h (L24, a24, and b24) at 35 days of age. 

The results presented in Table 5 reveal the effects of feed additives on meat characteristics, such as the Cooking Loss (CL), Water Holding Capacity (WHC), Myofiber Fragmentation Index (MFI), Shear Force (SF) and texture profile analysis (TPA) at 35 days of age under a *C. perfringens* challenge test. All treatment groups showed no significant differences concerning the CL, WHC, MFI, and SF. The T_6_ and T_5_ treatments exhibited significantly increased (*p* < 0.001) hardness and chewiness values compared with those of other treatment groups, respectively. Although the dietary supplementation showed no significant differences in Springiness and Cohesiveness values between all treated groups. 

## 4. Discussion

Necrotic enteritis (NE) has become one of the most critical problems in the poultry industry [50]. Feed additives, including antibiotics, prebiotics, and probiotics, have frequently been used for improving the health, growth, and feed efficiency parameters of animals [51]. Recently, interest in incorporating probiotics and antibiotics into broiler treatments has been rapidly increasing [52]. Several studies have shown that the addition of probiotics has positive effects on the growth rate, feed utilization, feed efficiency, and mortality rate [53,54]. However, the efficacy of probiotics depends upon the selection of more efficient strains, manipulation of genes, a combination of several strains, and the combination of probiotics and synergistically=acting components [55]. The use of multi-strain probiotics seems to be the best way of potentiating the efficacy of probiotics, as it beneficially affects the host by improving growth-promoting bacteria with competitive antagonism against pathogenic bacteria in the gastrointestinal tract [56].

### 4.1. Growth Performance 

The performance results obtained in the starter period and the finisher period are presented in Table 2. The IBW, TFI, BWG, and FCR of all groups receiving feed-supplemented diets and basal diets had no significant differences among them. However, the BWG, FBW, PEF, FCR, and SR during the finisher phase, at 15–35 days-old, were significantly (*p <* 0.05) improved in all supplemented diets compared to the control group. These results are similar to those of Khaksefidi and Ghoorchi [57], who found that the BWG of birds fed a diet supplemented with 50 mg/kg of *Bacillus subtilis* was significantly higher during the finisher period (22–42 d) than birds fed the control diets. Additionally, the feed conversion ratio of birds fed a diet supplemented with probiotics significantly reduced from 22 to 42 d compared with birds fed the control diets. Consequently, Patel et al. [55] indicated that the dietary supplementation of combined probiotics and prebiotics (Protexin) at 100 g/ton of feed significantly enhanced the BWG, along with an improved FCR, and benefitted without any adverse effects on the feed intake, mortality, or carcass characteristics. Also, Anjum et al. [58] and Singh et al. [59] observed similar results. The improvement of all performance parameters may be due to the biological role of probiotics in altering the intestinal pH, which modifies both the microbial population and nutrient absorption, ultimately improving the efficiency of feed utilization [60]. Moreover, increased feed intake and digestive enzyme secretions also are detected in animals’ fortified phytobiotic-supplemented feed [61]. Growth enhancement through the use of phytobiotics probably depends on the synergistic effects among complex active molecules present in phytobiotics [39,62].

The mortality rate in this study was low (finisher period), representing the positive effect of feed additives on the mortality rate (Table 2). These findings were similar to those found by Abdel–Hafeez et al. [63] and Riad et al. [64], who indicated that the addition of probiotics as feed additives decreased the mortality rate. The reduction in mortality was attributed to the inhibitory effects of these additives toward enteric microorganisms via modifying the intestinal pH [63]. Compared to a wide range of antibiotics (including Avilamycin), a significant decrease in mortality was seen for treated broilers with 2 g/kg Bio–Mannan-oligosaccharides (Bio–MOS) as a source of prebiotic, depending on the age of birds [65,66]. Moreover, there are major modes of action by which broiler performance is improved by proposed oligosaccharide prebiotics, such as control of type-1fimbriae pathogenic bacteria (mannose-sensitive lectin), immune modulation effects, and modulation of intestinal morphology and expression of mucin and brush border enzymes [67].

### 4.2. Carcass Traits

The mean values for the carcass characteristics, dressing percentage, and organ weight (%) relative to the bodyweight of the broilers are presented in Table 3. The dressing percentage was significantly (*p* < 0.05) increased in the T_6_ group compared with those of the other treatments. However, non-significant differences were observed in the other carcass measurements amongst the treatment groups. Some organ weight (spleen and thymus) significantly increased in probiotic supplemented diets alone or in combination with prebiotics compared with other treated groups. Generally, the inclusion of probiotics in broiler rations had no extra additional benefits for the organ weight or carcass yield [55]. Also, Salamkhan et al. [68], Panda et al. [69], and Patel et al. [55] reported that the dressing percentage did not differ significantly (*p* ≤ 0.05) between the probiotics-fed broilers and control broilers. To contrast, Banday and Risam [70] and Kabir et al. [71] observed a significant (*p* ≤ 0.05) improvement in the dressing percentage in groups supplemented with probiotics. Nevertheless, Sarangi et al. [72] reported a highly significant increment in breast yield in birds supplemented with prebiotics. Jin et al. [73] attributed the lower fat deposition in birds treated with probiotics to how the product could interfere in the availability of fat for lipogenesis in the birds.

### 4.3. Meat Quality Parameters

The effects of antibiotic-, probiotic-, and phytobiotic-supplemented diets on the measurements of meat characteristics under *C. perfringens* infection in broilers are presented in Table 4. The change in pH is one of the most significant changes that can occur during rigor mortis and can affect meat quality characteristics, such as texture, colour, and WHC [74]. Fletcher et al. [75] reported that post-mortem pH measurements are a good indicator of meat characteristics. Generally, a rapid post-mortem pH decline in breast meat can lead to protein denaturation, which may result in a pale colour and low WHC [76]. During this trial, the temperature and pH values obtained after slaughter and 24 h post-slaughter showed significant (*p* ≤ 0.05) enhancements for all supplemented diets compared with the group fed the basal diet. These results are similar to those found by Battula et al. [77] and Corzo et al. [78] where the bacterial challenge had negative impacts on the temperature and pH of the breast muscle.

Additionally, the cooking loses (CL) is a measure of the percentage of water lost during cooking as a result of shrinkage. Consequently, the degree of shrinkage upon cooking is directly correlated with a loss of juiciness. During this trial, the CL, WHC, and SF showed non-significant differences between the control and treatment groups. During a subsequent study, Pelicano et al. [79] examined the effects of *Lactobacillus-* and *Bacillus subtilis*-based probiotics on SF and reported non-significant values compared with the control group. However, the MFI of the breast muscle was influenced by the treatments (*p* ≤ 0.05), as shown in Table 4. The MFI decreased in T_2_ and T_3_ compared with the other treatment groups. Myofibrillar fragmentation is the extent of myofibril destruction caused by homogenization [80]. Olson and Stromer [81] reported that MFI values are correlated with other muscle indices, such as SF and tenderness. Therefore, it can be concluded that probiotic supplementation causes less damage to the myofibrils [76]. 

TPA is an instrumental measurement of the sensory attributes of chicken breasts which imitates the conditions to which food is subjected to in the mouth [76]. The TPA, which includes hardness, cohesiveness, springiness, and chewiness, is shown in Table 5. The treatments had no significant effects on any TPA variable (*p* ≤ 0.05). All tested groups were very tender, except for the T_6_ and T_5_ supplemented groups, which had hardness values that were significantly (*p* ≤ 0.001) higher than those of the other treatment groups. The data presented in this trial aligns with that of Angelovicova et al. [82] who noticed that probiotics (CloStat) moderately affect the hardness, cohesiveness, springiness, and chewiness of cooked breast meat. Also, Lisowski et al. [83] investigated a beneficial effect of the prebiotic on breast muscle weight and carcass percentage. It could be supposed on the one hand, that using prebiotics in broiler diets has a positive influence on muscle weight [84].

## 5. Conclusions

To conclude, fortified feed diets with 0.2, 0.5, 0.12 g kg^−1^ of *Bacillus subtilis* spores (Gallipro Tect), live *Bacillus* strain (CloStat) and phytobiotic natural compounds (Sangrovit Extra) respectively, alone or in combined form, could promote growth and reduce the NE mortality rates. Additionally, using these probiotic and phytobiotic compounds as an alternative for antibiotics in feed diets could be a useful strategy to ameliorate the harmful effects of *C. perfringens* bacterium on broilers, in terms of the performance, feed efficiency, meat quality and carcass characteristics regarding necrotic enteritis infections. 

## Figures and Tables

**Table 1:** Composition of starter and finisher diets.

Ingredient	Treatment Period (0‒35) Days	
	Starter (0–15)	Finisher (15–35)
Yellow corn	57.39	61.33
Soybean meal	27.00	22.80
Palm oil	2.20	2.80
Corn gluten meal	8.80	6.0
Wheat bran	0.00	3.0
Dicalcium phosphate (DCP)	2.30	2.09
Ground limestone	0.70	0.62
Choline chloride	0.05	0.05
DL-methionine	0.105	0.075
L-lysine	0.39	0.36
Salt	0.40	0.20
Threonine	0.17	0.17
V–M premix ^1^	0.50	0.50
Total	100	100
Analysis		
Metabolizable energy (ME) (kcal/kg)	3000	3050
Crude protein (%)	23.0	20.5
Non-phytate P (%)	0.48	0.44
Calcium (%)	0.96	0.88
Digestible lysine (%)	1.28	1.15
Digestible methionine (%)	0.60	0.54
Digestible sulfur amino acids (%)	0.95	0.86
Digestible threonine (%)	0.86	0.77

^1^ V–M premix; vitamin–mineral premix contains the following per kg: vitamin A, 2,400,000 IU; vitamin D, 1,000,000 IU; vitamin E, 16,000 IU; vitamin K, 800 mg; vitamin B1, 600 mg; vitamin B_2_, 1600 mg; vitamin B_6_, 1000 mg; vitamin B_12_, 6 mg; niacin, 8000 mg; folic acid, 400 mg; pantothenic acid, 3000 mg; biotin 40 mg; antioxidant, 3000 mg; cobalt, 80 mg; copper, 2000 mg; iodine, 400; iron, 1200 mg; manganese, 18,000 mg; selenium, 60 mg; zinc, 14,000 mg.

**Table 2:** Effects of some feed additives on the growth performance, survival rate, and intestinal lesion score of broilers challenged with *C. perfringens* during the starter and finisher feeding periods.

Measurements	Treatment						*p-*Value	
	T_1_	T_2_	T_3_	T_4_	T_5_	T_6_	SEM	Sig.
Starter Period (0–14 days-old)								
IBW (g)	36.7	36.9	36.8	36.8	36.9	36.8	0.04	NS
BW 14 d (g)	449	443.1	448	445.5	446.6	448.1	1.089	NS
TFI (g)	561.1	549.9	558.6	531.1	546.8	555.0	2.049	NS
BWG (g)	412.3	406.2	411.2	408.7	409.7	411.3	0.831	NS
FCR (g:g)	1.36	1.35	1.36	1.30	1.33	1.35	0.05	NS
PEF	189.0	206.4	189.2	217.0	213.8	209.0	1.049	NS
Finisher Period (15–35 days-old)								
FBW (g)	1593.3 ^b^	1807.4 ^a^	1827.0 ^a^	1824.3 ^a^	1823.1 ^a^	1798.9 ^a^	1.121	***
TFI (g)	2325.6	2366.7	2353.2	2382.6	2379.9	2404.4	2.865	NS
BWG (g)	1144.0 ^c^	1364.3 ^b^	1379.0 ^a^	1378.8 ^a^	1376.5 ^a^	1350.8 ^b^	2.961	***
FCR (g:g)	2.03 ^a^	1.73 ^bc^	1.71 ^c^	1.73 ^bc^	1.73 ^bc^	1.78 ^b^	0.03	***
PEF	215.6 ^c^	301.5 ^b^	305.0 ^ab^	310.0 ^a^	307.6 ^ab^	288.5 ^b^	3.76	***
SR (%)	97.2 ^b^	99.8 ^a^	99.6 ^a^	99.8 ^a^	99.8 ^a^	99.6 ^a^	0.30	***
Lesions score	2.50 ^a^	0.67 ^b^	0.66 ^b^	0.67 ^b^	0.33 ^c^	0.50 ^b^	0.26	***

IBW, initial body weight; BW 14 d, body weight at 14 days of age; FBW, final body weight; TFI, total feed intake; BWG, bodyweight gain; FCR, feed conversion ratio; PEF, protein efficiency ratio; and SR, survival rate. SEM, mean values of the standard error. Mean values of three replicates with deferent letters (^a^^,^^b,c^) in the same row are significantly different (*p* < 0.05). Sig, significance; NS, non-significance; *** significance at *p* < 0.001.

**Table 3:** Effects of some feed additives on the carcass traits of broilers challenged with *C. perfringens* during the starter and finisher feeding periods.

Measurements	Treatment						*p-*Valu*e*	
	T_1_	T_2_	T_3_	T_4_	T_5_	T_6_	SEM	Sig.
DP (%)^10^	65.7 ^b^	67.4 ^b^	67.3 ^b^	67.5 ^b^	67.4 ^b^	68.3 ^a^	0.65	**
Breast (%)^10^	24.8	25.6	24.9	26.2	25.6	25.8	0.94	NS
Leg (%)^10^	19.4	19.5	19.5	18.6	19.5	19.4	0.67	NS
Fat (%)^10^	1.35	1.25	1.37	1.08	1.30	1.45	0.18	NS
Liver (%)^10^	2.58	2.48	2.47	2.82	2.43	2.23	0.14	NS
Bursa (%)^10^	0.17	0.19	0.16	0.15	0.20	0.19	0.02	NS
Spleen (%)^10^	0.11 ^ab^	0.11 ^ab^	0.09 ^b^	0.12 ^ab^	0.13 ^a^	0.10 ^b^	0.09	**
Thymus (%)^10^	0.52 ^a^	0.49 ^ab^	0.57 ^a^	0.42 ^c^	0.49 ^ab^	0.45 ^b^	0.04	*
Gizzard (%)^10^	3.52	2.85	2.90	3.08	3.38	3.10	0.21	NS
Heart (%)^10^	0.53	0.45	0.48	0.60	0.57	0.50	0.04	NS

DP, dressing percentage. SEM, mean values of the standard error. Mean values of three replicates with different letters (^a,b,c^) in the same row are significantly different (*p* < 0.05). Sig, significance; NS, non-significance; *, significance at *p* < 0.05; **, significance at *p* < 0.01.

**Table 4:** Effects of some feed additives on the meat quality of broilers challenged with *C. perfringens* during a feeding trial period.

Measurements	Treatment						*p-*Value	
	T_1_	T_2_	T_3_	T_4_	T_5_	T_6_	SEM	Sig.
pHi	6.58 ^ab^	6.63 ^a^	6.52 ^b^	6.59 ^ab^	6.55 ^b^	6.60 ^ab^	0.03	**
pHu	6.00 ^b^	6.07 ^a^	6.05 ^ab^	6.05 ^ab^	5.93 ^c^	5.94 ^c^	0.02	***
Temperature (℃)	27.82 ^ab^	28.43 ^a^	28.98 ^a^	27.03 ^ab^	26.10 ^b^	24.84 ^c^	0.32	***
L^*^_15_	42.29	45.22	45.56	44.11	44.40	44.38	0.93	NS
a^*^_15_	6.32	4.78	6.09	7.26	5.37	4.97	0.64	NS
b^*^_15_	2.34	2.68	2.68	1.91	2.23	1.73	0.55	NS
L^*^_24_	52.69	52.70	52.63	53.35	53.84	53.60	0.81	NS
a^*^_24_	5.11	5.85	5.57	6.75	5.51	5.76	0.60	NS
b^*^_24_	6.57	5.77	5.58	5.52	7.50	6.50	0.71	NS

pHi, meat pH after slaughter; pHu, meat pH after 24 h; Temp., carcass temperature after slaughter; L15, a15, and b15, colour components after slaughter; and L*24, a*24, and b*24, colour components after 24 h. SEM, mean values of the standard error. Mean values of three replicates with different letters (^a,b,c^) in the same row are significantly different (*p* < 0.05). Sig, significance; NS, non-significance; **, significance at *p* < 0.01; ***, significance at *p* < 0.001.

**Table 5:** Effects of some feed additives on the meat characteristics of broilers challenged with *C. perfringens* during a feeding trial period.

Measurements	Treatment						*p*-Value	
	T_1_	T_2_	T_3_	T_4_	T_5_	T_6_	SEM	Sig.
CL%	25.98	26.85	30.56	29.65	29.38	28.87	1.61	NS
WHC	0.25	0.31	0.28	0.29	0.28	0.28	0.02	NS
MFI	108.43	90.70	91.27	110.55	109.12	110.67	5.14	NS
SF (kg)	1.26	1.54	1.44	1.44	1.40	1.40	0.18	NS
Texture Profile Analysis (TPA)								
Hardness (kg)	1.09 ^b^	0.69 ^c^	0.71 ^bc^	1.21 ^ab^	1.49 ^a^	1.51 ^a^	0.12	***
Springiness	0.58	0.61	0.62	0.59	0.58	0.60	0.02	NS
Cohesiveness	0.53	0.51	0.53	0.52	0.50	0.52	0.01	NS
Chewiness	0.34 ^b^	0.22 ^c^	0.24 ^c^	0.37 ^ab^	0.43 ^a^	0.47 ^a^	0.04	***

CL, cooking loss; WHC, water holding capacity; MFI, Myofiber Fragmentation Index; SF, shear force; and TPA, texture profile analysis. SEM, mean values of the standard error. Mean values of three replicates with different letters (^a^^,^^b,c^) in the same row are significantly different (*p* < 0.05). Sig, significance; NS, non-significance; ***, significance at *p* < 0.001.
